# Is Life Binary or Gradual?

**DOI:** 10.3390/life14050564

**Published:** 2024-04-27

**Authors:** Christophe Malaterre

**Affiliations:** 1 Département de Philosophie, Université du Québec à Montréal, Montreal, QC H3C 3P8, Canada; malaterre.christophe@uqam.ca; 2 Centre Interuniversitaire de Recherche sur la Science et la Technologie (CIRST), Montreal, QC H3C 3P8, Canada

**Keywords:** life definitions, binary nature of life, gradualist perspective, lifeness, aliveness, grayness of life, epistemic granularity, epistemic perspective, threshold

## Abstract

The binary nature of life is deeply ingrained in daily experiences, evident in the stark distinctions between life and death and the living and the inert. While this binary perspective aligns with disciplines like medicine and much of biology, uncertainties emerge in fields such as microbiology, virology, synthetic biology, and systems chemistry, where intermediate entities challenge straightforward classification as living or non-living. This contribution explores the motivations behind both binary and non-binary conceptualizations of life. Despite the perceived necessity to unequivocally define life, especially in the context of origin of life research and astrobiology, mounting evidence indicates a gray area between what is intuitively clearly alive and what is distinctly not alive. This prompts consideration of a gradualist perspective, depicting life as a spectrum with varying degrees of “lifeness”. Given the current state of science, the existence or not of a definite threshold remains open. Nevertheless, shifts in epistemic granularity and epistemic perspective influence the framing of the question, and scientific advancements narrow down possible answers: if a threshold exists, it can only be at a finer level than what is intuitively taken as living or non-living. This underscores the need for a more refined distinction between the inanimate and the living.

## 1. Introduction

Life, a concept deeply resonant in our daily experiences, holds a privileged place within biology—a discipline explicitly dedicated to the study of living organisms. Defining life has perennially been a quintessential philosophical endeavor. Across centuries, thinkers have proposed myriad conceptions, mirroring the complexity and richness inherent in this fundamental notion. In antiquity, Aristotle linked life to the possession of a soul, establishing a connection between life and the inner dimension of existence [1]. Leibniz, in his Monadology, posited that life resulted from the aggregation of monads, indivisible units of existence [2]. Buffon, at the dawn of modern biology, viewed life as an inherent property of organic molecules, while Kant defined it as the presence of organized bodies [3,4]. The diversity of perspectives underscores the challenge of capturing the complex and ever-evolving nature of life. Today, this conceptual quest persists—a testament to an enduring philosophical challenge [5]. Scientists such as Schrödinger [6], Oparin [7], Crick [8], de Duve [9], Maynard-Smith and Szathmáry [10], among many others, have sought to define life by enumerating specific properties: material exchange, individual growth, self-reproduction, evolution, metabolism, thermodynamic disequilibrium, and more. While these definitions often present refinements, many can be perceived as highlighting certain perspectives, such as metabolic or genetic considerations [11]. Despite criticisms questioning the very feasibility of defining life [12,13,14], in practice, researchers frequently revert to definitions like NASA’s, which asserts that “life is a self-sustained chemical system capable of undergoing Darwinian evolution” [15,16]. Indeed, given the state of the art, such definitions are deemed adequate, at least as working definitions [17], and a pragmatic consensus tends to converge around them, with the understanding that revisions will be introduced as evidence and theory demand.

Irrespective of the nuanced details within these definitions, which can be informative and valuable in their own right, a crucial observation emerges—they generally portray life as a binary construct, embodying a dichotomy between the living and the non-living [18]. This conceptualization inevitably gives rise to the identification of thresholds, occasionally situated at varying points, as is, for instance, the case with definitions that accentuate individual and collective properties in disparate manners [19,20,21,22]. While one might contend that this poses no issue if the definitions are interpreted not as tools for classification but rather as serving alternative purposes like operational or theoretical objectives [17,23,24,25], the crux of the matter lies in the inherent nature of these definitions. Regardless of their intended function, they can always be wielded as classificatory tools. This even appears to be an intrinsic outcome of the very act of defining, which inherently seeks precise demarcations akin to the identification of necessary and sufficient conditions.

The paradoxical nature of the discussion becomes apparent when contemplating life definitions that often draw clear boundaries, positing a dichotomy between the living and non-living, yet simultaneously recognizing a continuum of intermediates, both in contemporary science and likely during the historical origin of life on Earth [5,11]. As a matter of fact, this dichotomy of life does not always withstand close scrutiny, particularly when delving into specific fields of science that probe the frontiers of life [26]. In microbiology, for instance, an increasing number of microscopic entities such as giant viruses and reduced microbial symbionts are found that defy clear classification, illustrating the inherent grayness of life forms [27,28]. In synthetic biology and systems chemistry, artificial entities are synthesized that challenge our traditional views of life [29,30,31]. As a result, a more nuanced approach, grounded in a gradualist conception of life, has emerged [32]. This perspective acknowledges different degrees of life that characterize a gray area between the inert and the living. Concepts like “lifeness signatures”, “lifeness space” [33], and “aliveness” [34,35] have been proposed to account for this idea of gradation.

This presents a dilemma. On one hand, there is an evident necessity to define life for clarity and consensus; however, the methods of definition, often specifying necessary and sufficient conditions, tend to depict life as a binary concept. On the other hand, mounting evidence points toward a gray zone between what is unmistakably alive and what is distinctly not alive. The fundamental question persists: Is life inherently binary, or does it exist on a gradual or gray spectrum?

This question holds significant implications across metaphysical, epistemic, and methodological dimensions relevant to the practice of science. At its metaphysical core, the debate delves into the essential nature of life, questioning the ontological continuity or discontinuity between living and non-living entities and touching on broader philosophical considerations about whether life holds a special status in our ontology of nature. Epistemic implications concern our description and understanding of life as afforded by the best available scientific knowledge. Fueled by empirical investigation, the debate directly shapes how we conceptualize and theorize about life, with binary perspectives enforcing distinct boundaries between the living and non-living, while gradual views suggest a nuanced spectrum challenging traditional perspectives. Methodological implications impact fields such as astrobiology and origin of life research. While the search for extraterrestrial life typically relies on binary assumptions, a gradual perspective notably prompts a reconsideration of search criteria and the adoption of a more flexible approach, accommodating a broader spectrum of potential conditions. As for research on the experimental synthesis of life, a gradual perspective allows for a more intricate exploration of possible intermediate states between living and non-living entities.

This contribution seeks to explore the foundational motivations behind interpreting life as either binary or non-binary. Acknowledging the presence of a gray zone inhabited by structurally and functionally diverse entities, the imposition of a threshold for defining life is called into question. Unless there is compelling evidence of a definitive transition threshold between what is unmistakably alive and what is unequivocally not alive, any demarcation point for life becomes conventional, if not arbitrary. Consequently, the pivotal question revolves around whether there exist valid reasons to perceive life as a binary phenomenon or as a gradual spectrum. The contribution suggests that answering this question depends significantly on how it is framed, particularly in terms of epistemic granularity (level of detail) and epistemic perspective (choice of descriptive space or variable space). However, evidence constrains assertions within a specific granularity and a specific perspective. If a definitive threshold exists, it must be situated at a much finer granularity than is often presupposed in intuitive understandings of life and non-life.

The remainder of the essay unfolds as follows: Section 2 describes what seems to be an obvious aspect—the binary nature of life, according both to common sense and to science. Section 3 raises doubts about the validity of this binary perspective. Section 4 demonstrates how this dichotomy falters in the practice of astrobiology. Section 5 elaborates on what it means to consider life in degrees. Section 6 delves into a potential objection—underdetermination—and responds with two counterarguments: epistemic granularity and epistemic perspective. Section 7 addresses a second objection, that of relativism, by examining the knowledge garnered to date by science. Finally, Section 8 concludes by emphasizing the importance of identifying fine-grained degrees of life for astrobiology, and notably for origin of life studies.

## 2. The Perceived Binary Nature of Life

Definitions that enumerate sets of characteristics deemed necessary and sufficient to characterize life, or that present themselves as models to which living systems should somehow be isomorphic, go hand-in-hand with the binary presupposition that in nature, two sets of objects exist—those that are alive (corresponding to the adopted definition of life) and those that are not. Following [36], the binary presupposition of life can be formulated as follows:

*(Bi)* For any entity *E*, *E* is either alive or not alive.

This binary presupposition is widely embraced by common sense and what can be called the folk concept of life [37]. It is reasonable to think that it finds justification in everyday life and in the classification of the various entities that surround us. *(Bi)* is indeed mobilized in two contrasting contexts that delineate life along two distinct oppositions.

The first, a more immediate and intuitive contrast, stems from the intrinsic human desire to distinguish entities that are alive from those that are no longer so. This life-death opposition holds profound significance, facilitating, for instance, the emotional processes of mourning, the identification of potential threats, or the release from future responsibilities towards a deceased entity. It tends to apply diachronically to the same entity, marking a before and after, influencing personal perceptions of life’s transience and related emotional responses.

The second contrast delineates the living from the inert, framing life against non-life. Unlike the first opposition, this distinction is not centered on a change in an entity’s state but rather aims to categorize diverse entities based on their inherent nature. The practical usefulness of this contrast is manifold. Classifying entities based on whether they are alive or inert helps identify those that may pose a potential danger: a bear does not pose the same danger as a rock (at least to a certain extent). It can also serve to delineate responsibilities, as in the case of certain environmental theories, such as biocentrism, where specific duties are theorized towards living entities. This second opposition tends to operate synchronically, classifying various entities according to their inherent characteristics.

The binary presupposition of life finds validation in common experiences that confirm the validity of these oppositions. As such, it is deeply embedded in daily life, influencing personal narratives of life, death, and the distinctions between the animate and the inanimate.

In the sciences, these two oppositions also play pivotal roles in grounding binary conceptions of life. Many health-related disciplines, including medicine and veterinary sciences, inherently consider life in opposition to death. Other disciplines presuppose a distinction between the living and the inert. Thus, biodiversity research seeks to inventory living entities and their species, while astrobiology-contributing disciplines such as astronomy or synthetic biology, respectively, seek to identify or create the living in opposition to the inert.

Thus, the binary presupposition *(Bi)*, deeply rooted in common sense, aligns with scientific practices. It suggests that classifying entities into two distinct groups is often relevant. However, the question persists: Is this binary framework truly reflective of the intricacies inherent in the diverse manifestations of life? Does the binary principle *(Bi)* stand up to scrutiny? Are the life-death and living-inert oppositions as robust as they seem?

## 3. Challenges to the Binary Nature of Life

Despite endless confirmations, the life-death opposition, even in everyday practice, is not always straightforward to implement. Pronouncing an individual’s death can be extremely challenging, leading to debates on the appropriate criteria to choose for determining a state of brain death or neurological death. It is noteworthy that an individual’s brain death can coexist with the preservation, for a certain duration, of a form of metabolic life, enabling the harvesting of organs that are still alive and well functioning. Even more challenging is the possibility of brain death reversal, as recently investigated [38,39]. The transition from life to death thereby appears much less distinct than the life-death opposition suggests in the first place, hinting at a reality more intricate than *(Bi)* implies.

A closer look reveals that the living-inert opposition is also occasionally challenged and of no practical use. In biodiversity research, for instance, the opposition is frequently non-operational despite patent usage of the term “bio”: studies often emphasize macroorganisms (e.g., animals, plants) at the expense of microorganisms (e.g., bacteria), thereby excluding a substantial portion of what is living. For instance, the executive summary of the IPBES—the Intergovernmental Science-Policy Platform on Biodiversity and Ecosystem Services—2019 report does not mention microorganisms, bacteria, or viruses—although they are acknowledged in the full report, as they should [40]. Another example is ecosystem restoration, which commonly focuses on plants and animals [41,42]. Paradoxically, some diversity studies may examine non-living entities or entities classified as such, as seen in research on viral diversity [43].

Moreover, microbiology introduces numerous entities that challenge classification, casting doubt on our very intuitions about life and non-life. This includes viruses whose size and genomic complexity surpass those of some of the smallest bacteria [27,44] and bacteria so functionally and genetically reduced that their true living nature is questioned [28,45]. The microbiological menagerie also encompasses artificially simplified versions of natural organisms [46], RNA viral particles or viroids [47], viral agents known as satellites [48,49], virus of viruses known as virophages [50], autonomous DNA strands or plasmids [51], prions [52], and many more.

Reality thereby appears far more intricate than the life-death and living-inert oppositions suggest. A similar complexity arises in astrobiology, as we shall now explore.

## 4. One or Multiple Lives in Astrobiology?

Astrobiology encompasses various disciplines and a diversity of methodological approaches, converging around the broadly framed question of the origin of life and its potential existence beyond Earth. This is evident in the programmatic definition of the European Astrobiology Roadmap (AstRoMap), which construes astrobiology as “the study of the origin, evolution, and distribution of life in the context of cosmic evolution; this includes habitability in the Solar System and beyond” [53]. This also shows in the axiological perspective of the NASA astrobiology roadmap, according to which “astrobiology embraces the search for potentially inhabited planets beyond our Solar System, the exploration of Mars and the outer planets, laboratory and field investigations of the origins and early evolution of life, and studies of the potential of life to adapt to future challenges, both on Earth and in space” [54]. As a consequence of this breadth of scope, astrobiology brings together numerous scientific disciplines with diverse approaches, providing complementary perspectives on the origins and distribution of life in the broadest sense. These approaches and associated research themes are not merely programmatic but are evident in scientific practice, as shown by analyses of scientific corpora, including thematic text-mining analyses of key journals in the field [55].

Amidst this diversity of perspectives, two approaches (among others) can be identified where the distinction between living and inert entities appears relevant while simultaneously giving rise to slightly different interpretations or, at the very least, different research targets related to the identification of the presence of life. The first focuses on the synthesis of living systems. This approach is typically found in the fields of synthetic biology and systems chemistry, encompassing research programs aligned or not with the prebiotic conditions of early Earth. These programs typically employ as starting material a broad set of compounds, be they prebiotically plausible molecules, macromolecules extracted from current living entities, or artificially synthesized chemical compounds. In these research endeavors, a focal objective is the synthesis of proto-living systems, essentially designing life in the laboratory. The idea is to experimentally demonstrate the feasibility of bridging the gap between the inert and the living by conceiving chemical systems, albeit minimal, capable of possessing the major functionalities typically attributed to clearly living entities (e.g., [29,30,31,56]). The goal is thereby to create chemical systems that start to resemble something alive but in a minimal sense—so to speak, minimally living systems.

A different approach where the living/inert distinction appears relevant is the search for traces of life or “biosignatures” (e.g., [57,58,59,60]). This search can be conducted in the context of examining ancient terrestrial rocks to potentially identify extremely ancient traces of life, as performed within the domains of geology and paleobiology. The search for biosignatures also extends to space exploration and the quest for signs of life elsewhere in the solar system, such as Mars or the icy moons of Jupiter or Saturn. Additionally, it occurs in the discovery and characterization of an increasing number of exoplanets, particularly through the meticulous examination of the atmospheric spectra of these planets. In all of these cases, the objective is to sift through available evidence to identify empirically compelling elements affirming a biological origin—essentially, that a genuinely living entity is the source of what we observe. By doing so, such biosignatures implicitly refer to a conception of life that is already quite sophisticated, for instance, a life capable of metabolism so specific that it leaves an indisputable trace, impossible to confuse with abiotic processes.

While the first case targets, in practice, minimally living entities, the second case implicitly involves undeniably living entities capable of producing convincing traces of life. This difference also shows in the type of epistemic risk that each approach seeks to mitigate. While the first case likely wishes to avoid the non-detection of minimal life synthesized in the laboratory—false negatives—in controlled, replicable conditions designed to eliminate any false positives, the second case conceives the epistemic risk in terms of false positives: the goal is to avoid affirming the existence of life where there is none, which would otherwise be detrimental.

In any case, these two approaches have research targets based on different conceptions of life, thus allowing for the consideration of a spectrum of degrees of life, ranging from minimally living systems as pursued by synthetic biology to undeniably living systems as typically presupposed by the search for biosignatures.

## 5. Conceptualizing Life in Degrees

All the above challenges to sorting entities into distinct categories—with regards to their status as being alive or not—tend to indicate that life is more a matter of degrees than a clear-cut, unequivocal transition. If this is the case, then the presupposition *(Bi)* of the binary nature of life is not as strongly justified as sometimes advocated, at least when examined with sufficient detail or precision. Life would be more about “more or less” than “all or nothing,” with borderline cases between two well-identified extremes: the clearly non-living and the clearly living (at least from what we call tell, which is to say epistemically speaking). Here, we encounter an old idea already present, for example, in the works of Pirie [61], regarding the existence of a gray or indeterminate zone between what is clearly alive and what is clearly not alive. This leads to adopting a gradualist assumption, that can be formulated as follows:

*(G)* For any entity *E*, *E* is either clearly alive, clearly not alive, or more or less alive between these two extremes.

One way to formalize this intermediate zone of degrees of life (“lifeness space” or zone of “aliveness”) is to affirm the existence of a graded scale between the clearly non-living at point 0 (such as elementary molecules, e.g., CO_2_, NH_3_...) and the clearly living at point 1 (represented by sophisticated unicellular organisms undeniably alive to our eyes, e.g., well-studied bacteria such as *E. coli* or *M. genitalium*) [32,33,35,62].

The question then arises, assuming prior agreement on benchmarks for the clearly not alive and the clearly alive states, of measuring such degrees of life for entities of the in-between zone. A straightforward way to measure such degrees of life would be to assess, as a whole, the extent to which the activities of these entities relate to the activities of specific clearly living entities (e.g., metabolizing components, multiplying, producing variation, etc.) in contrast to clearly non-living entities (e.g., possessing a mass, just like living entities).

Although this solution may seem simple at first, the problem is that more-or-less living entities may potentially perform very different activities from one another. For instance, a giant virus may store a vast amount of genetic information but cannot metabolize all the components necessary for its replication alone, explaining incidentally its crucial ability to interact with other clearly living organisms for that purpose in particular. Conversely, a reduced endosymbiotic bacterium may store less genetic information while still capable of synthesizing a relatively large part of its components, although far from achieving autonomy and limited in its interactions with its host bacterium only.

A solution to the problem of measuring lifeness is to identify what these entities do—meaning, to identify their various activities or functional capacities—and measure, comparatively, their degree of performance in carrying out these activities. Indeed, while it might be challenging to compare the overall lifeness of entities performing very different activities, it seems easier to compare their activities one at a time. Consider, for example, the capacity for individuation (i.e., the structures and processes by which an entity distinguishes itself from its surrounding environment).

Individuation can be achieved in various ways and, accordingly, be given different performance scores. One can conceive a rudimentary mode of individuation in mineral compartments (e.g., [63]); such compartments would provide means of individuation but would be fixed, with little opportunity for directed molecular exchanges with the environment. A slightly more sophisticated mode of individuation is that of bilayer vesicles resulting from the self-assembly of simple amphiphilic molecules (e.g., fatty acids); although flexible and mobile, these vesicles remain very fragile, sensitive to concentration, temperature, and pH [64]. A slightly more performant mode of individuation would be obtained by inserting into these membranes stabilizing molecules such as sterols or amphiphilic polypeptides, resulting in more robust vesicles [65,66]. One can imagine that a membrane equipped with specialized transporters and active catalysts would constitute an even more sophisticated mode of individuation, capable of creating and maintaining chemical imbalances with its environment (e.g., [67]). Up to the extremely sophisticated and multifunctional membranes of some current microorganisms, such as those serving as benchmarks for degree 1 of life.

**Figure 1:**
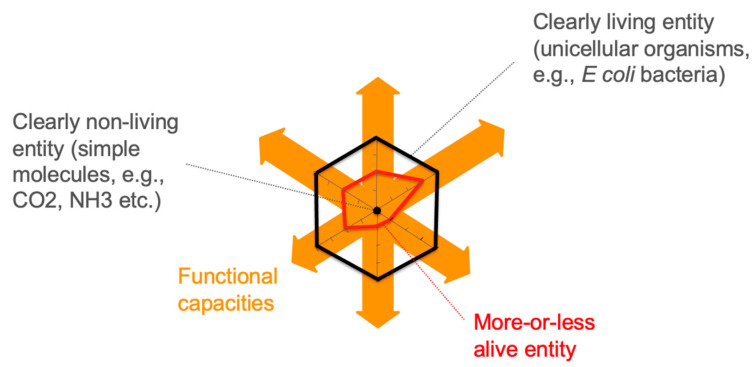
Lifeness space and lifeness signatures. Hallmark functional capacities of benchmark living entities define a lifeness space. Functional performance is maximum (1) for benchmark clearly living entities, and minimal (0) for benchmark clearly non-living entities. The varying performance of a more-or-less alive entity along the different capacities defines its specific lifeness signature.

Once similar performance evaluations are completed, for a given entity, along with all the hallmark activities or functional capacities associated with the benchmark of clearly living entities, they define a multidimensional “lifeness signature” (Figure 1). Within this lifeness space, any entity is represented by a vector whose distance from the 1-mark hyperplane indicates its proximity to be as alive as clearly alive entities. This vector representation facilitates meaningful comparisons between entities while making it possible to average these measures per entity and determine an overall degree of life for each.

A possible operationalization of such a measure of lifeness suggests that a legitimate way to identify both the functional capacities of entities and their degree of performance (or sophistication) is to analyze the scientific discourse about these entities [36]. The underlying hypothesis is that what science says about these entities is the best source of information about their functional capacities. Identifying the functional capacities of an entity then involves identifying the themes present in the scientific discourse about that entity. The more a theme is present and correlated with a given entity, the more it can be said to depict a specific and sophisticated functional capacity for that entity. Analyzing this discourse with text-mining methods applied to extensive corpora of scientific articles makes it possible to identify these functional capacities, resulting in a multidimensional performance vector or “lifeness signature”. When assessing lifeness, reaching the 1-mark is what matters, although one might consider other contexts where measuring performance beyond this 1-mark could be pertinent (for instance, if it becomes relevant to distinguish between various types of clearly living entities). This multidimensional representation can also be expanded to include other relevant dimensions (for instance, if the discovery of extraterrestrial life departs from life-as-we-know-it and pushes us to revise these dimensions).

It becomes evident that it is not only possible but also more empirically adequate to describe the gray zone between what is clearly non-living and what is clearly living as populated by more-or-less living entities. These entities have globally less performant life signatures than clearly living entities. Depending on whether one adopts a research perspective aiming to characterize the diversity of life today or during its emergence from inert matter on the primitive Earth, the entities populating the lifeness space will either be existing entities today, identified, for example, by microbiology, or entities identified as possible intermediaries in a process of emergence and evolution of life.

This results in an empirical argument against the binary principle *(Bi)* of life. It establishes the feasibility of identifying degrees of life, thus reinterpreting the abrupt threshold stipulated by *(Bi)* into a gradual transition as accommodated by *(G)*. Of course, this presupposes seeking to characterize life in more detail, with a finer epistemic granularity, so to speak.

## 6. Objection of Underdetermination and Response

A possible objection to this gradualist perspective may appeal to the argument of underdetermination, attempting to weaken the justificatory foundations of the conception of life in degrees by emphasizing that this conception empirically relies so to speak on a limited number of data points. Despite the various examples mentioned earlier, relatively few entities have been discovered, and even fewer have been artificially synthesized to date that could be classified in this gray area between the clearly non-living and the clearly living. The empirical basis of the gradualist argument is therefore relatively sparse, leaving room for the possible existence of a significant threshold yet to be uncovered somewhere amidst these few scattered points. By redefining the boundaries of this hypothetical threshold as demarcations between the inanimate on one side and the clearly living on the other, it becomes possible to reintroduce the binary principle. This objection can be articulated in two ways: one might argue that a possible threshold could exist along one or possibly several dimensions of the lifeness space; alternatively, it could be argued that such a threshold might affect all lifeness dimensions at once.

**Figure 2:**
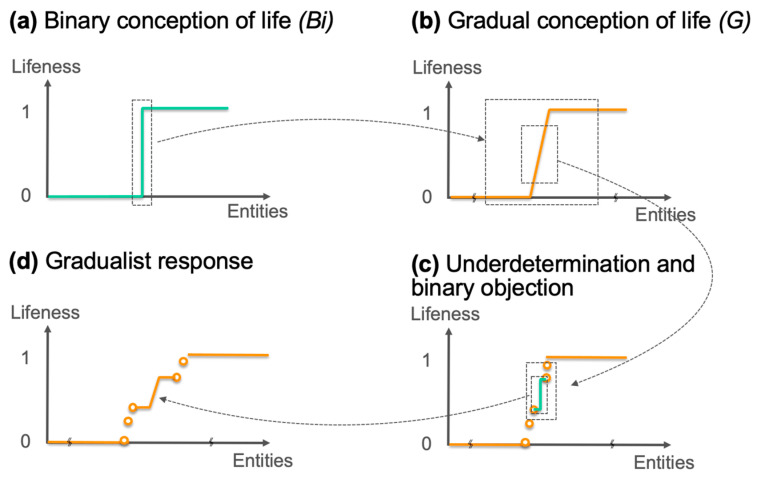
Possible argument dynamics between binary and gradual conceptions of life. Graphs depict overall lifeness as a function of entity type (sorted by e.g., complexity). Similar representations could be made that focus on just a single relevant dimension of lifeness. (**a**) According to *(Bi)*, a clear threshold separates life from non-life (in green). (**b**) A gradual conception *(G)* argues distinctions in lifeness exist at a finer epistemic granularity (in orange). (**c**) A new threshold is posited at an even finer granularity (in green). (**d**) Gradual distinctions in lifeness are reintroduced at yet a finer granularity (in orange).

In any case, this objection involves adopting a finer epistemic granularity than that adopted by the gradualist thesis, allowing for the identification of possible spaces between certain groups of empirical data points and reintroducing the possible existence of a major threshold in one of these spaces based on a principled argument (Figure 2).

A response to this objection would involve similarly crafting a principled argument invoking yet another change in epistemic granularity. Indeed, nothing in principle forbids to conceive of the possible existence of novel more or less alive entities whose lifeness might fit between the bounds of this new alleged life threshold. One could even argue that since this heuristic has already proven successful, leading to the identification of more or less living entities like the ones discussed above, it is likely to work again and result in the identification of new entities between these previously identified ones, especially within the new threshold proposed by the objection to the gradualist argument (Figure 2d). In sum, while a gradual transition might be criticized as possibly housing a threshold transition at a finer grain, a threshold transition might in turn prove to be more gradual at an even finer grain.

It is easy to understand that the objection to the gradualist argument and the response it elicits are principled points that may give rise to an infinite regression, continually motivated by changes in increasingly finer epistemic granularity. There are at least two ways to stop the regression. The first is to argue that epistemic granularity, which ultimately concerns the detail or finesse of describing the phenomena of interest, is actually contextual, meaning that it depends on a research context specifying the required level of detail. Thus, if a certain level of detail is required for a specific question, another level of detail may appear more relevant given another research question. In essence, this is to say that arbitrations are possible between levels of granularity, all equally legitimate depending on the pursued research objective. This implies that both *(Bi)* and *(G)* can be recognized as valid or relevant but at different levels of granularity, given different epistemic contexts.

The second way to address this problem of a possible infinite regression is to acknowledge that if there is a threshold, it can only be now at a finer level of granularity than initially envisaged by *(Bi)*. Indeed, the existence of a certain number of more or less living entities discovered by science (such as giant viruses or micro-endosymbiotic bacteria as discussed above) constrains any threshold, if it exists, to position itself at best between some of these entities. The consequence is that a new binary hypothesis *(Bi’)* must be formulated, with its narrower bounds being some of these gray entities. Science indeed provides descriptions with increasingly finer granularity, somehow closer to reality. The discoveries of more or less living entities act as constraints on *(Bi)*, pushing toward reformulations mobilizing finer-grained bounds that are increasingly narrower from what intuitively might be termed clearly non-living and clearly living.

**Figure 3:**
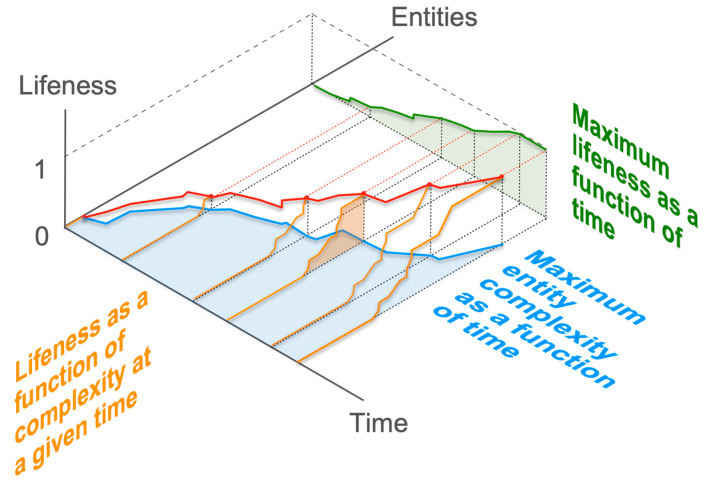
Epistemic perspectives on *(Bi)* and *(G)*. Binary or gradualist arguments can be deployed by considering lifeness distribution over entities (sorted by e.g., complexity) at a given time (in orange), by considering maximum lifeness reached by the most complex entities over time (in green), maximum entity complexity over time (in blue), or maximum lifeness as a function of time and maximum entity complexity (in red). A threshold according to one perspective may reveal a gradual transition according to another. Note: for simplification, types of entities that appear through time are assumed to be more complex than previous ones and to persist (orange curves are cumulative over time); more realist representations would show changes in the lifeness curves as a function of complexity, accounting for novel entities at all values of lifeness and extinctions.

Another element to consider is the epistemic perspective that is adopted when advancing *(Bi)*, where the epistemic perspective refers to the angle of describing a phenomenon (which is reflected notably in the choice of descriptive variables). Indeed, it is entirely possible that a threshold transition according to one perspective might turn out to be gradual according to another perspective. In the case at hand, the epistemic perspective seeks to characterize the existence or non-existence of a major lifeness threshold capable of distinguishing entities in the world. This perspective presupposes that, at a given point in time, all existing entities are somehow listed and sorted in a principled way, for example, in ascending order of complexity—or with another metric such as assembly theory [68]—and then that their lifeness is evaluated. Whether *(Bi)* or *(G)* are true should then be seen on the plot of this lifeness as a function of entity complexity ranking, either showing significant threshold transitions or gradual ones (Figure 3, orange plots).

Yet, one can also seek to characterize how the maximum level of lifeness changes over time. This perspective is particularly relevant when considering the historical question of the origin of life. The descriptive variables change compared to the first, synchronic case: we now have to consider the plot of maximum lifeness over time (Figure 3, green plot), a perspective that can fuel questions about underlying evolutionary processes [69,70,71,72,73,74,75]. One can also be interested in the evolution of the maximum degree of complexity through time, which is yet another perspective on the overall same class of phenomena (Figure 3, blue plot). The key point is that what appears as a threshold transition according to one perspective may turn out to be a gradual transition according to another perspective. For example, what may appear as a threshold transition according to a synchronic perspective (existence of a lifeness threshold between certain entities at a given moment) may prove to be the result of a gradual perfection of a given type of entities over time (or even an increase in lifeness associated with the disappearance of intermediate entities).

**Figure 4:**
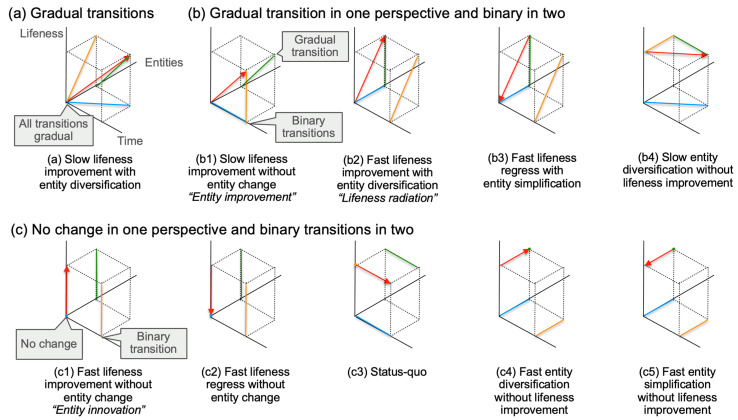
Binary and gradual transitions depending on epistemic perspective. (**a**) The generic case where all transitions appear gradual, independently of which perspective is chosen; this represents the case of slow lifeness improvement with entity diversification resulting in increased complexity. (**b**) Cases that depict a gradual transition in one perspective and binary transitions in the other two, representing different types of lifeness improvement patterns with or without entity diversification and complexification. (**c**) Cases with no apparent change in one perspective and binary transitions in two, depicting other patterns of change, including status-quo. Actual trajectory of maximum lifeness as a function of time and maximum entity complexity represented in red, with an arrow indicating the direction of change; projections into the different perspectives (planes) in orange (lifeness as a function of complexity at a given time), green (maximum lifeness as a function of time), and blue (maximum entity complexity as a function of time) (same color coding as Figure 3).

Note that taking into account these different epistemic perspectives allows for distinguishing between several types of transitions that may take place at a relatively fine granularity (Figure 4). For instance, some form of “entity improvement” might be at work when a relatively slow improvement of lifeness takes place without entity change (Figure 4(b1)). A “lifeness radiation” may take place when a sudden increase in lifeness occurs together with the appearance of numerous entities (Figure 4(b2)). What can be termed “entity innovation”, may consist of a fast lifeness improvement without any change in entity type (Figure 4(c1)).

In any case, the choice of perspective is crucial for assessing the binary or gradual character of any transition. Just by considering these three dimensions (lifeness, entities sorted by complexity, and time), the relevance of *(Bi)* or of *(G)* is immediately questioned across three possible projection planes, each representing a unique epistemic perspective. Importantly, what might appear as a threshold transition in one perspective could manifest as a gradual transition, or even no discernible change, from another perspective. Recognizing that the state-space of entities with varying degrees of lifeness encompasses dimensions beyond the three discussed, the existence of numerous threshold or gradual transitions is anticipated. For origin of life research, a critical consideration is understanding not only how each threshold is traversed but also how different thresholds and gradual transitions might be interconnected or linked [76]. This interconnectedness is essential for bridging the gap between inanimate and living matter.

## 7. Objection of Radical Relativism and Response

Turning the binary or non-binary nature of life into a question relative to both granularity and epistemic perspective might run the risk of facing criticism for radical relativism: if every judgment about the nature of life is relative to a given granularity and a given descriptive perspective, it would imply that, ultimately, no answer to the question could be provided. It would suggest that determining the nature of the living and its specific continuity or discontinuity with the non-living is impossible.

Several reasons prompt us to resist this criticism. The first is to assert that just because a conclusion about the question is relative to epistemic granularity and perspective does not mean it does not exist. There is every reason to believe, on the contrary, that a particular characterization of life is adequate precisely given the context in which the question is posed, especially considering the epistemic granularity and perspective that are adopted. In other words, once epistemic granularity and perspective are fixed, there is every reason to believe that an answer can be provided to the question, and it can turn out to be empirically adequate and useful in that epistemic context. Certain contexts may require, for very pragmatic reasons, to draw a clear line within the gray zone between life and non-life, and may even do so in different ways.

As we saw (Section 4), exoplanetologists and synthetic biologists do not need to mean the same exact thing by “life” in order to carry out their research: in their respective contexts, certain aspects of life will be relevant and not others. This aligns with a pragmatic view according to which certain vague concepts—such as “function” and others—may be disambiguated differently depending on context [77]. In this respect, certain entities will be alive in certain contexts and not in others, since whether one uses this line or that line, or no line at all, certainly is determined by pragmatic elements of the context. Yet, at the same time, the degree of lifeness for any given type of entity remains an objective fact of the world: this is something that ultimately can be measured independently of those contextual perspectives. Similarly, whether that degree of lifeness falls on this side or that side of a given line in the gray zone is also a set fact. As a result, while this gray zone of lifeness aligns with Wittgensteinian family resemblance views or blurry property cluster kinds theses [78,79,80], not everything goes: measuring lifeness introduces a principled way to empirically map out this gray zone, allowing us to speak in meaningful terms about the relative differences of the entities that are found in between its bounds.

The second reason to resist the criticism of radical relativism is to acknowledge the incremental progress made by science. These advancements gradually populate our abstract theoretical landscape with new empirical data points that act as constraints on our conceptualization of the world. The identification of new, more or less alive entities in microbiology, virology, and molecular biology, as mentioned earlier, is a significant first step. In origin of life research, two other types of work are particularly relevant to this question.

The first involves research into the possible existence of a minimal genome. This is, in a sense, a top-down approach to the question of the origin of life. Starting from clearly living entities, this work aims to identify a much-reduced set of genes compared to the initial genomes but still allowing for a minimal form of life in organisms possessing them. Some of this work is theoretical and benefits from phylogenetic and comparative genomic approaches [81,82]. Other approaches are empirical and can rely on knockout experiments of target genes deemed non-essential in laboratory-studied organisms or on the artificial synthesis of a reduced genome [46,83]. With such research, the threshold of the “clearly living” (mark 1 of lifeness), previously associated with organisms like the bacterium *E. coli*, for which its lifeness status is beyond doubt, now encounters other marks with a lifeness certainly still very high but less than that of *E. coli*. Faced with this state of affairs, two options present themselves: either we can change the benchmark organism and now turn to an organism with a minimal genome, or we can assign to such an organism a lifeness degree slightly lower than 1. In the first case, this means changing our intuitions about the types of organisms that can be categorized as clearly living. In the other, it means acknowledging the existence of new entities less clearly alive than those previously known, and thus pushing the possible existence of a threshold *(Bi)*—if it exists—down to a lower level (and therefore a finer epistemic granularity).

The second type of work that can provide insights into novel more or less alive entities includes bottom-up initiatives stemming from prebiotic chemistry, systems chemistry, synthetic biology, or molecular biology. The idea here is to start from clearly non-living chemical or biochemical compounds (even if sometimes extracted from living organisms) to try to create systems with some of the key capabilities of living entities. However, these systems often exhibit relatively weak performance compared to what living entities are currently capable of. These include, for instance, systems containing small autocatalytic networks capable of amplifying to some extent or encapsulated systems in liposomes capable of growth and division [29,30,31]. Understandably, such systems, even if they do not possess all the functional capacities of a clearly living organism like *E. coli*, and even if they are not as efficient as *E. coli* for the functional capacities they possess, deserve to be categorized as having a lifeness higher than that of simple molecules taken as benchmarks for the inanimate (mark 0).

Overall, these two types of research indicate that if the question of the existence of a specific threshold delineating the transition between the inanimate and the living persists, as per *(Bi)*, it no longer does so in the same terms: any potential threshold, if it exists, might only exist between lifeness marks now slightly above 0 on one side and slightly below 1 on the other. However, its boundaries will no longer align with the initial benchmarks for the clearly living and the clearly non-living entities.

If we do not want to constantly change benchmarks for the limits of 0 and 1 of lifeness—which is wise to maintain a common basis for comparison as science advances—this means giving up *(Bi)* as initially formulated and falling back onto a new formulation *(Bi’)* taking into account these novel empirical lifeness points. In other words, the advances of science—at least in the two domains of origin of life research just discussed (top-down and bottom-up), but also in microbiology, virology, or molecular biology as mentioned earlier (Section 3)—constrain the possible position and scope of a binary transition, and narrow it down to benchmark points that are not as further apart as the initial benchmark points were. Thus, not everything is relative to granularity and epistemic perspective: scientific advancements determine empirical constraints that tend to make certain conceptions of the boundary between the inanimate and the living more plausible. If some sort of lifeness threshold exists, then it can only be at a finer scale than initially stipulated by *(Bi)*.

## 8. Conclusions: The Importance of a Finer Grained Perspective

The binary nature of life appears self-evident in everyday discourse, justified by the repeated contrasts between life and death, living and inanimate, experienced in our daily lives. It also finds justification in certain scientific disciplines, such as medicine and biology. However, doubts may seriously arise about the truly binary nature of life. This holds true for common sense when judging the living or non-living status of certain entities, but it is especially evident in science. This is notably the case in microbiology, virology, and molecular biology, where new boundary entities are discovered that are challenging to classify as clearly non-living or clearly living. Similarly, in astrobiology and origin of life studies, the coexistence of different conceptions of what is targeted as a life phenomenon (strong conception in the search for biosignatures, weak in synthetic biology and systems chemistry) casts doubts on the existence of an unequivocal threshold. Without empirical identification of a precise transition threshold, any delineation point for life remains conventional.

Given the uncertainty surrounding the existence of a definitive threshold, contemplating a gradual distinction between entities that are unmistakably non-living and those that are undeniably living becomes a sensible approach. Within this gradualist perspective, life is viewed as existing on a spectrum or exhibiting degrees of “lifeness” tied to the possession and varying efficiency of specific functional capacities. This conceptual framework allows for the characterization of boundary entities based on their distinct “lifeness signatures”, acknowledging the existence of intermediates between non-living matter and living matter.

Certainly, objections can be raised against this gradualist conception, citing its underdetermination, which leaves room for the possible existence of a threshold transition at a finer granularity. Nevertheless, two responses are possible: (1) a new change in epistemic granularity could identify new intermediate entities and restore a gradual conception; (2) a change in epistemic perspective (e.g., adding the temporal dimension) may identify a gradual transition behind a threshold transition.

While criticism may be levied, these responses do not open the door to radical relativism. Indeed, scientific advances bring constraints and already allow us to discard certain formulations of the binary assumption of life *(Bi)*. In particular, if the existence or non-existence of a major threshold remains open, it can only be at a finer granularity than intuitive conceptions of life and non-life. This underscores the importance of seeking to characterize these degrees of life more precisely. It seems that there is more to gain in terms of heuristics and the precision of the descriptive discourse of nature than in affirming the existence of an irreducible threshold. A finer conception of the inanimate-living distinction, as precisely proposed by the gradualist conception, would be highly beneficial for certain scientific domains, such as certain research programs in systems chemistry or synthetic biology, which specifically require a finer granularity to characterize the objects they work on.

## Data Availability

No new data were created or analyzed in this study. Data sharing is not applicable to this article.
